# The feasibility of an intensive case management program for injection drug users on antiretroviral therapy in St. Petersburg, Russia

**DOI:** 10.1186/1477-7517-10-15

**Published:** 2013-09-05

**Authors:** Alla V Shaboltas, Roman V Skochilov, Lillian B Brown, Vanessa N Elharrar, Andrei P Kozlov, Irving F Hoffman

**Affiliations:** 1 St. Petersburg St. University, Universitetskaya nab. 7/9, St.Petersburg, Russia; 2 Department of Medicine, University of North Carolina, Manning Drive, Chapel Hill, NC, USA; 3 NIAID, NIH, 6700-B Rockledge Drive, 5125, Bethesda, MD 20892, USA; 4 The Biomedical Center, Vyborgskaya st. 8, St.Petersburg, Russia

**Keywords:** Injection drug users, Russia, HIV/AIDS, Antiretroviral therapy, Case management

## Abstract

**Background:**

The majority of HIV-infected individuals requiring antiretroviral therapy (ART) in Russia are Injection Drug Users (IDU). Substitution therapy used as part of a comprehensive harm reduction program is unavailable in Russia. Past data shows that only 16% of IDU receiving substance abuse treatment completed the course without relapse, and only 40% of IDU on ART remained on treatment at 6 months. Our goal was to determine if it was feasible to improve these historic outcomes by adding intensive case management (ICM) to the substance abuse and ART treatment programs for IDU.

**Methods:**

IDU starting ART and able to involve a “supporter” who would assist in their treatment plan were enrolled. ICM included opiate detoxification, bi-monthly contact and counseling with the case, weekly group sessions, monthly contact with the “supporter” and home visits as needed. Full follow- up (FFU) was 8 months. Stata v10 (College Station, TX) was used for all analysis. Descriptive statistics were calculated for all baseline demographic variables, baseline and follow-up CD4 count, and viral load. Median baseline and follow-up CD4 counts and RNA levels were compared using the Kruskal-Wallis test. The proportion of participants with RNA < 1000 copies mL at baseline and follow-up was compared using Fisher’s Exact test. McNemar’s test for paired proportions was used to compare the change in proportion of participants with RNA < 1000 copies mL from baseline to follow-up.

**Results:**

Between November 2007 and December 2008, 60 IDU were enrolled. 34 (56.7%) were male. 54/60 (90.0%) remained in FFU. Overall, 31/60 (52%) were active IDU at enrollment and 27 (45%) were active at their last follow-up visit. 40/60 (66.7%) attended all of their ART clinic visits, 13/60 (21.7%) missed one or more visit but remained on ART, and 7/60 (11.7%) stopped ART before the end of FFU. Overall, 39/53 (74%) had a final 6–8 month HIV RNA viral load (VL) < 1000 copies/mL.

**Conclusions:**

Despite no substitution therapy to assist IDU in substance abuse and ART treatment programs, ICM was feasible, and the retention and adherence of IDU on ART in St. Petersburg could be greatly enhanced by adding ICM to the existing treatment programs.

## Background

Political, economic, and social changes accompanying the fall of the Soviet Union in the 1990’s contributed to the establishment of opium-based drug trafficking routes from Afghanistan through Russia and a rapid increase in injection drug abuse rates [1]. Russia currently has one of the highest rates of injecting drug users (IDU) in the world at 1.8% among adults over 15 years of age [2] and it is estimated that one of the highest IDU populations in the world (> 80,000 active IDU) live in St. Petersburg, the second largest city in Russia [3]. Unsafe injection drug practices drive the HIV epidemic in Russia, including St. Petersburg; 76% of incident HIV cases were associated with unsafe injection practices [4]. In addition, among HIV infected women who have never injected drugs, almost half have had sex with an IDU [5]. HIV prevalence and incidence rates among IDU in St. Petersburg have been consistently high, with a 30% prevalence and an incidence of 4.5 per 100 person-years in 2003 [6,7], 14 per 100 person-years incidence in 2008 [8], and 35% prevalence and 7.2 per 100 person-years incidence in 2010 [9].

Few IDU in Russia are currently receiving anti-retroviral treatment (ART). Almost 90% of all people with HIV in Russia are IDU but only 6% on ART are IDU [10]. One of the main obstacles to drug abuse and HIV treatment of active IDU is the low adherence and retention rates. Only 16% of IDU who attended substance abuse treatment programs completed the entire course without relapse [11]. In Russian the following components are available as substance abuse treatment: short-term intensive detoxification, opiate antagonist treatment (naltrexone), psychotherapy and social rehabilitation. The majority of IDU in St.Petersburg have access only to short-term detoxification, counseling with psychotherapy elements and social rehabilitation programs based on the 12-step model. Although HIV care and ART are currently available free for all HIV-infected individuals according to WHO guidelines for starting ART, self report and cross-sectional studies suggest IDU are not only less likely to access ART, they are less likely to be retained in care: only 40% of IDU on ART remain on treatment at 6 months [12]. Thus clinicians remain reluctant to treat IDUs because they fear low retention and adherence [13]. There is currently no effective system of case management for persons on ART in Russia.

Adherence to ART is critical to the effective treatment of HIV/AIDS. Adherence to prescribed doses may need to be as high as 90%-95% to achieve suppression of viral replication and prevent the development of resistant viral variants [14]. Active substance abuse has been associated with a decreased adherence to ART [15-17]. However, former users or those in substance abuse therapy programs have comparable adherence to those that never used [18]. IDU status itself is not a barrier to treat HIV. A meta-analysis of 12 studies (>9000 patients) found no difference in ART resistance rates among IDU (23% of sample) and non-IDU [19].

Case management is an effective strategy for HIV infected IDU patients to improve substance abuse and HIV treatment outcomes [20,21]. HIV-infected individuals with case managers are more likely to receive benefits advocacy, psychological services, and emotional and practical support. HIV case management is associated with increased utilization of support services and a decrease in unmet needs [22,23]. A brief, focused case management system in U.S. urban centers helped newly diagnosed HIV-positive individuals successfully access HIV care [24]. Among HIV-infected homeless and marginally-housed individuals, case management was associated with improved self-reported antiretroviral adherence and increased CD4 cell count [25]. A system of case-management of HIV-infected IDU in Brazil was widely acceptable to health care professionals involved in the medical care of IDU and peer-based support groups contributed to increased ART adherence [26]. In Lesotho a nurse-initiated managed care system resulted in a retention in care rate of 80% at 12 months and 77% at 24 months [27], and case management has resulted in adherence of greater than 80% in Mozambique and Brazil [28,29]. The inclusion of informal social networks, community support and relatives or friends in care is shown to be beneficial [30-32].

A prospective cohort study of HIV uninfected IDUs at risk for HIV-infection in St. Petersburg (DAIDS HPTN 033) demonstrated the effectiveness of a case management model in retention of IDU to long-term follow-up [6,7]. This study obtained 80% retention, and when adjusted for non-controlled reasons, such as incarceration or death, the retention rate was 90%. This clearly demonstrates the potential for intensive case management (ICM) to improve retention among active IDUs. We sought to examine the feasibility of ICM to improve substance abuse and HIV treatment outcomes for IDU in Russia.

## Methods

### Study population, inclusion criteria

HIV-infected, active IDU (within the last 6 months), who were eligible to start ART or recently began ART (within the last 3 month); were able to identify a parent, relative, partner or friend (supporter) who could actively assist them in their treatment plans; and for the active users, were willing to enroll in a 10 day in-patient detoxification program, were recruited at the City AIDS Center, St. Petersburg, Russia and invited to participate in the feasibility study.

### Study visits

Once an IDU was confirmed eligible, an enrollment visit was conducted which included an informed consent procedure, a risk assessment questionnaire including drug use and psychological status assessment, an HIV risk behavior evaluation, a physical examination of the skin looking for fresh needle marks, a urine drug screen and an alcohol breathalyzer. Enrollment also included a baseline CD4 count and HIV RNA viral load result extracted from the patients HIV care chart. If the participant was not currently on ART, ART began within 30 days of the enrollment visit. In addition, the IDU provided detailed locator information for themselves and their identified supporter.

Each participant was assigned to a personal case management team: a social worker and psychologist who both were responsible for the patient’s follow-up and the development and implementation of their individual case management plan. Phone contacts or home visits were conducted by case managers when any ART clinic appointment was scheduled or missed. Any scheduled appointment was considered a missed visit after one week without re-scheduling through communication between the case manager or City AIDS Center staff and the patient. The case managers also communicated monthly with the patient supporter or more frequently during any crisis period or to assist with a scheduled or missed appointment.

Follow-up study visits occurred every 2 weeks for the 8 months of study duration. The full follow-up period was determined per protocol and was set at 8 months due to budget restrictions. Each follow-up visit included an interview on recent behavior including drug use, self reported adherence to their ART regimen, social harms, a physical exam looking for fresh needle marks, and a urine drug test. Also during each follow-up visit participants received individual drug and ART counseling. ART clinic appointment schedules (and ART distribution) were variable, depended on the clinician, and ranged from every one month to every three months. During visits at 4 and 8 months the HIV risk behavior and psychological behavior questionnaire was repeated.

### Outcomes

Retention was stratified into three categories based on how many months of ART each participant received: 1. attended all clinic appointments including the 8 month follow-up visit; 2. attended some but not all clinic appointments, including the 8 month follow-up visit; 3. stopped ART before the 8 month follow-up period was complete. Adherence was determined by the final HIV RNA result that occurred between their 6–8 month follow-up visit.

HIV viral load was quantified using Roche RNA PCR 1.5 with a lower limit of detection of 50 copies/mL. CD4 was quantified using FACS count, both conducted as the standard of care at the City AIDS Center.

Drug abuse relapse was considered three consecutive days of drug use after a negative urine drug screen and was determined by self-report, history provided by the supporter, clinical examination for the presence/absence of fresh puncture marks and additional urine drug tests. Case management was continued regardless of drug use status.

### Analysis

Descriptive statistics were calculated for all baseline demographic variables, baseline and follow-up CD4 count, and viral load. Median baseline and follow-up CD4 counts and RNA levels were compared using the Kruskal-Wallis test. The proportion of participants with RNA < 1000 copies mL at baseline and follow-up was compared using Fisher’s Exact test. McNemar’s test for paired proportions was used to compare the change in proportion of participants with RNA < 1000 from baseline to follow-up. Stata v10 (College Station, TX) was used for all analysis.

### Ethical considerations

This pilot program was conducted in compliance with the protocol, International Conference on Harmonization Good Clinical Practice E6 (ICH-GCP) and the applicable regulatory requirements and the ethical considerations stated in the declaration of Helsinki. This program was approved by institutional review boards at the University of North Carolina and the Biomedical Center in St. Petersburg.

## Results

Between November 2007 and May 2008, 901 HIV positive patients at the City AIDS center in St. Petersburg, Russia were screened for inclusion in the pilot study. Among them 346 (38.4%) were injection drug users with a history of active injection in the past 6 months, and 60 (17.3%) were eligible and consented to participate. The primary reasons for ineligibility were 171 (49.4%) were not eligible for ART, 49 (14.2%) refused to attend detoxification and only 16 (4.6%) had no supporter. Among the 60 enrolled, 34 (56.7%) were male; the median age was 31 years (range 18–41); 46 (76.7%) had at least a secondary education and only 7 (11.7%) were fully employed. The median age at first drug injection was 17 years with a median of 10 years of abuse. The “supporters” were mostly female (51; 85.0%); a parent (37; 61.7%); or a sexual partner (14; 23.3%).

At enrollment 31/60 (51.7%) were actively injecting. 29/60 (48.3%) had actively injected in the previous 6 month but were drug free at the enrollment date. Among the 31 actively injecting participants 30 (96.8%) were injecting heroin, 1 (3.2%) was injecting psycho stimulants, and 21/31 (67.7%) admitted to sharing injection paraphernalia in the past month. All 31 active users began a 10-day detoxification program but only 5 (16.1%) completed the program. However, 26/31 (83.9%) repeated the detox program at least one additional time during their 8-month follow-up period.

All 60 subjects started on ART between 3 months prior to enrollment and 1 months following enrollment. Sixteen (26.7%) started ART prior to enrollment. The reasons for ART initiation were 45 (75.0%) CD4 <300 cells/mL; 31 (51.7%) RNA >50,000 copies mL; and 3 (5.0%) an opportunistic infection. 28/60 (46.7%) were asymptomatic at initiation. The initial ART regimens included 29 (61.7%) AZT/3TC/EFV; 7 (14.9%) AZT/3TC/LPV/r; and 4 (8.5%) DDI/3TC/NVP.

The overall follow-up rate with the case managers at 8 months was 54/60 (90.0%). 40 subjects (66.7%) attended all of their ART clinic appointments (Group 1); 13 (21.7%) were partially compliant to their ART clinic appointments (Group 2), including 6/13 (46%) who attended at least 90% of their clinic appointments; and 7 (11.7%) stopped attending their appointments (and receiving ART) on their own prior to the end of the 8 month follow-up period (Group 3) (Table 1). There was no statistically significant difference between the 3 retention groups according to gender, age, education, employment or living situation (data not shown).

**Table 1:** ART follow-up and response (N = 60)

	**N**	**Median number of participant ART clinic visits**	**Median number of weeks in follow-up**
Participants who received ART the entire 8 months	40 (66.7%)	5	36
Participants who were partially adherent to ART during 8 months	13 (21.7%)	4	34
Participants who stopped ART before 8 months	7 (11.7%)	1	21

**Figure 1:**
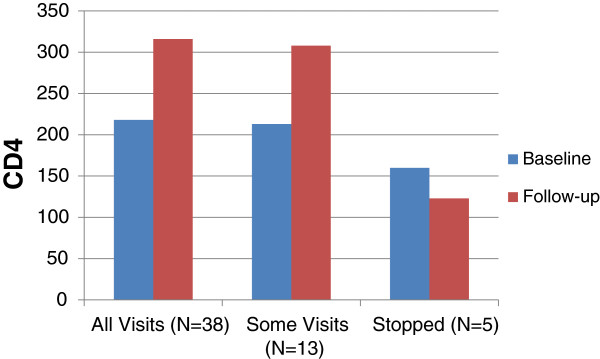
**CD4 at Baseline and last follow-up visit.** Figure 1 provides data on baseline (ART initiation) and last follow-up CD4 result by levels of retention. At initiation the total median CD4 was 215 cells/mL with the median among the group who stopped ART at 160 cells/mL. There was no difference in the median CD4 count between the three groups at baseline (p = 0.33). There was a statistically significant improvement in the CD4 count at the last follow-up visit compared to the baseline visit for the groups that were completely or partially adherent, but not for the group that stopped ART prior to the 8 month visit.

**Figure 2:**
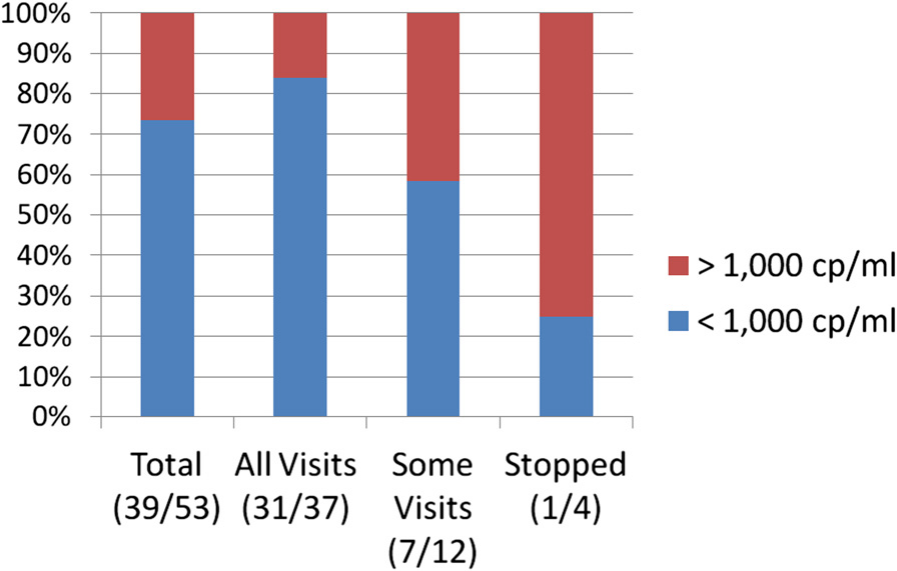
**HIV RNA at last follow-up visit.** Figure 2 provides data on ART initiation and last follow-up viral load by levels of retention. The total median viral load at initiation was 12,000 copies/mL with the medians per adherence group ranging from 5,842 to 341,259 copies/mL with the highest value in the group that stopped ART early. At the last follow-up visit, the overall median RNA copies/mL was 64, with a median range from 50 copies/mL in those who attended all clinic visits to 3,750 in those who stopped ART prior to the end of the follow-up (p = 0.04). 84% of the fully adherent group had RNA copies mL <1000; the partially adherent group had 58% and the group that stopped ART only 25% (p = 0.02).

The 8 month intensive case management effort included a medium number of 15 case manager contacts per subject, of which 33.4% were unscheduled contacts and the result of missed appointments or personal crisis. In addition, during the 8 month follow up period the case managers had a medium of 8 contacts with each “supporter” of which 18.2% were also unscheduled. Group counseling sessions were offered on a weekly basis. However, only 24/60 (40%) attended at least one session and the median number of sessions attended by these 24 was 2. Table 2, and Figures 1 and 2 provide data on the ART initiation and last follow-up CD4 and RNA result by levels of retention. At initiation the total median CD4 was 215 cells/mL with the median among the group who stopped ART at 160 cells/mL. There was no difference in the median CD4 count between the three groups at baseline (p = 0.33). There was a statistically significant improvement in the CD4 count at the last follow-up visit compared to the baseline visit for the groups that were completely or partially adherent, but not for the group that stopped ART prior to the 8 month visit. The total median viral load at initiation was 12,000 copies/mL with the medians per adherence group ranging from 5,842 to 341,259 copies/mL with the highest value in the group that stopped ART early. At the last follow-up visit, the overall median RNA copies/mL was 64, with a median range from 50 copies/mL in those who attended all clinic visits to 3,750 in those who stopped ART prior to the end of the follow-up (p = 0.04). 84% of the fully adherent group had RNA copies mL <1000; the partially adherent group had 58% and the group that stopped ART only 25% (p = 0.02). There was no demographic or behavioral difference by group between the participants who provided values to the CD4 and RNA analysis and those who did not.

**Table 2:** CD4 and RNA at initiation and at 8 month (or last visit) by levels of ART adherence (N = 60)

	**Attended all clinic visits (n = 40)**	**Attended some clinic visits, including 8 month visit (n = 13)**	**Stopped ART before 8 month visit (n = 7)**	**Total (n = 60)**
CD4 Count				
Initiation CD4 Count: n(%)	38 (95%)	13 (100%)	5 (71%)	56 (93%)
Median (IQR)	219 (145,306)	213 (84,323)	160 (124,216)	215 (129,301)
Follow-up CD4 Count: n(%)	38 (95%)	13 (100%)	5 (71%)	56 (93%)
Median (IQR)	316 (194,384)	308 (113,360)	123 (100,253)	293 (154,382)
	*p* = 0.02	*p* = 0.03	*p* = 0.5	
Viral Load (VL)				
Initiation VL: n(%)	35 (88%)	12 (92%)	4 (57%)	51 (85%)
RNA <1000: n(%)	16 (46%)	4 (33%)	1 (25%)	21 (41%)
Median (IQR)	5840 (61,156000)	7440 (114,872779)	341259 (75232,911102)	12000 (75,268000)
Final VL: n(%)	37 (93%)	12 (92%)	4 (57 %)	53 (88%)
RNA <1000: n(%)	31 (84%)*	7 (58%)*	1 (25%)*	39 (74%)
	p < 0.001	p = 0.4	p = 1.0	
Median (IQR)	50 (50,400)**	275 (50,7022)**	3750 (907,153000)**	64 (50,1030)

*p = 0.02 **p = 0.04.

Overall, 31/60 (51.7%) were active IDU at enrollment and 27 (45.0%) were active at their last follow-up visit. However, among the 29 (48.3%) subjects who were drug free at enrollment, 7 (24.1%) relapsed and were active users at their last visit. Overall, 33/60 (55.0%) were drug-free at the last follow-up visit.

**Figure 3:**
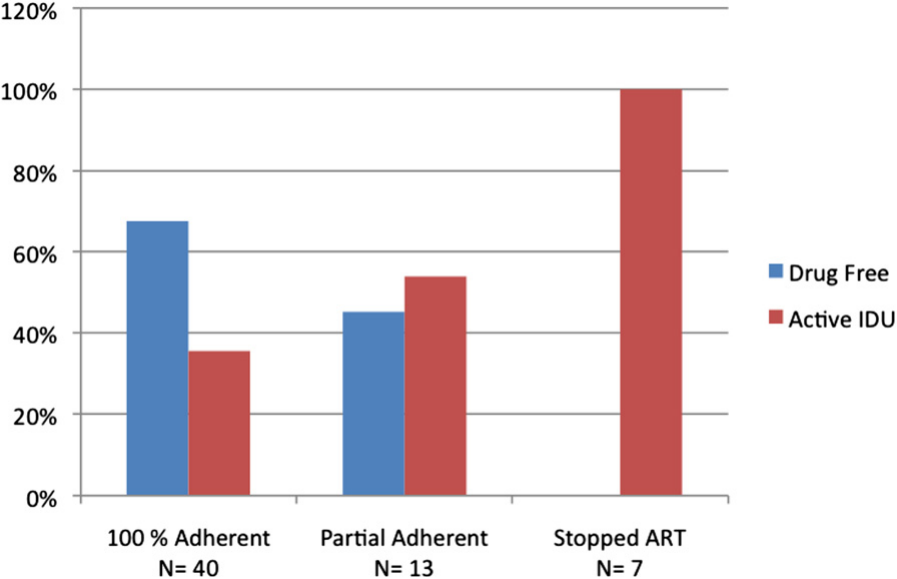
**ART adherence and IDU status at last follow-up visit.** Figure 3 illustrates the IDU status (drug free or active) of the subjects at their last follow-up visit stratified by their ART clinic appointment attendance. Being drug free was associated with better adherence/retention where 27/40 (67.5%) of the group with 100% clinic attendance were drug free, 6/13 (46.2%) of the group that attended most, but not all of their visits were drug free, and none 0/7 (00.0%) of the group that stopped ART and their clinic appointments before the end of the 8 month follow-up period were drug free (P = 0.002).

Figure 3 illustrates the IDU status (drug free or active) of the subjects at their last follow-up visit stratified by their ART clinic appointment attendance. Being drug free was associated with better adherence/retention where 27/40 (67.5%) of the group with 100% clinic attendance were drug free, 6/13 (46.2%) of the group that attended most, but not all of their visits were drug free, and none 0/7 (00.0%) of the group that stopped ART and their clinic appointments before the end of the 8 month follow-up period were drug free (P = 0.002).

## Discussion

Intensive case management for HIV infected IDU is feasible and can be an effective complement to improve HIV treatment outcomes, including retention and adherence in Russia. The majority (90%) of participants enrolled in this pilot study remained in research follow up until the end of the 8 month project period and 74% had a viral load <1000 copies/mL at their last visit. This is a significant improvement compared to historic data where only 40% of IDU starting on ART in St. Petersburg were retained in care at 6 months, and comparable to the ART adherence performance in the US where, over a ten year period, 78% of patients achieved viral suppression 6 months after starting combination ART [33]. Although our feasibility study had a small number of participants, we have shown that with intensive case management, IDU who have a social or family support system can achieve high levels of ART adherence (74%) and retention in care (88%) despite continued or unstable drug use. Although we limited our enrollment to IDU who identified a support person to help them with their treatment program, only 5% of the 346 IDU’s screened did not have a viable support person. This is reassuring and consistent with the demographic data that shows almost all IDU either live at home with their parents or have a steady sexual partner. Thus, the strategy of including such a support person in case management treatment planning could be scaled up.

Because no substitution therapy is available in Russia, the options for substance abuse treatment are limited. Short-term detoxification is notoriously ineffective and psychological counseling without replacement therapy also has an extremely high relapse rate. Naltrexone, an opioid antagonist reduces relapse in Russia [11,34] but is very expensive and not available to most injectors, especially in the public sector. Overall, we observed a modest reduction in active drug use, however one quarter of IDU who were not actively injecting at enrollment relapsed during the follow-up, reflecting the reality of drug addiction in the absence of either substitution therapy or naltrexone. Indeed, all the participants who were lost to follow-up or stopped ART before the end of the follow-up period were actively injecting. Countries where substitution therapy is not available for opiate dependent HIV-infected persons requiring ART pose a significant challenge to effective treatment, mostly due to poor adherence and retention. Even among our population of injectors who all had a social support system, intensive case management and the provision of antiretroviral therapy, there was virtually no effect on drug use.

The HIV/AIDS epidemic in Russia parallels the epidemic of drug use in Russia. The majority of participants in our cohort began injecting drugs when they were less than 20 years of age and have now been IDUs for at least 10 years. Many of this initial cohort of drug users who are still alive, now require ART. However, the success of the ART program will be hampered by continued drug use and no widely accessible and effective substance abuse treatment program.

A comprehensive, multidisciplinary approach to care for HIV infected IDU has proven effective in other settings [35-37]. In Brazil, an integrated system of mobile case management and ART and primary care treatment at the same location as substance abuse treatment including substitution therapy was successfully implemented [26]. Integrating office-based opioid dependence treatment in HIV primary care has been promoted as an effective method to improve treatment for HIV-infected drug users [38].

The limitations of our feasibility study include the small numbers of IDUs followed, no real- time comparison group that limited our ability to determine effectiveness, and questions about scaling up such a labor intensive case management system. Though feasible for a limited number of IDU, its unlikely that the resources would be available to scale up such an intensive program. A less intensive case management intervention would be sustainable and scalable. We believe the “supporter” support system could be retained within this system, but we do not know how fewer case manager contacts with the case and their supporter would affect the outcomes. Enhancing the use of cell phone technology would be one way to reduce the cost per case managed.

## Conclusions

Overall, intensive case management for IDU on ART is a feasible and promising strategy to enhance substance abuse and ART treatment in Russia. A case management system provides both individual and public health opportunities for prevention activities. Comprehensive prevention packages that coordinate substance abuse and HIV prevention strategies using case management as the cornerstone for HIV positive and HIV negative IDU, with appropriate replacement therapy, will be essential to improving substance abuse and HIV outcomes.

## Competing interests

None of authors have financial or non-financial competing interests.

## Authors’ contributions

AVS contributed to the study design, coordinated all study procedures and drafted the manuscript. RVS participated in the study design, data collection and statistical analysis. IFH contributed to the study design, protocol development, data analysis and writing the manuscript. LBB participated in statistical analysis and preparation of the first draft of manuscript. VNE contributed to data analysis and logistic. APK contributed to logistic and study management. All authors read and approved the final manuscript.

## References

[B1] Kalichman SC Kelly JA Sikkema KJ Koslov AP Shaboltas AV Granskaya JV The emerging AIDS crisis in Russia: review of enabling factors and prevention needs Int J STD AIDS 2000 11 2 71 75 10.1258/0956462001915345 10678472

[B2] Mathers BM Degenhardt L Phillips B Wiessing L Hickman M Strathdee SA Wodak A Panda S Tyndall M Toufik A Mattick RP Global epidemiology of injecting drug use and HIV among people who inject drugs: a systematic review Lancet 2008 372 9651 1733 1745 10.1016/S0140-6736(08)61311-2 18817968

[B3] Heimer R White E Estimation of the number of injection drug users in St. Petersburg, Russia Drug Alcohol Depend 2010 109 1–3 79 83 20060238 10.1016/j.drugalcdep.2009.12.010 PMC2875272

[B4] Gyarmathy VA Li N Tobin KE Hoffman IF Sokolov N Levchenko J Batluk J Kozlov AA Kozlov AP Latkin CA Injecting equipment sharing in Russian drug injecting dyads AIDS Behav 2010 14 1 141 151 10.1007/s10461-008-9518-6 19214731 PMC2818991

[B5] Toussova O Shcherbakova I Volkova G Niccolai L Heimer R Kozlov A Potential bridges of heterosexual HIV transmission from drug users to the general population in St. Petersburg, Russia: is it easy to be a young female? J Urban Health 2009 86 Suppl 1 121 130 19533368 10.1007/s11524-009-9364-5 PMC2705483

[B6] Kozlov AP Shaboltas AV Toussova OV Verevochkin SV Masse BR Perdue T Beauchamp G Sheldon W Miller WC Heimer R Ryder RW Hoffman IF HIV incidence and factors associated with HIV acquisition among injection drug users in St Petersburg, Russia. AIDS 2006 20 6 901 906 10.1097/01.aids.0000218555.36661.9c 16549975

[B7] Shaboltas AV Toussova OV Hoffman IF Heimer R Verevochkin SV Ryder RW Khoshnood K Perdue T Masse BR Kozlov AP HIV prevalence, sociodemographic, and behavioral correlates and recruitment methods among injection drug users in St. Petersburg, Russia J Acquir Immune Defic Syndr 2006 41 5 657 663 16652041 10.1097/01.qai.0000220166.56866.22

[B8] Niccolai LM Verevochkin SV Toussova OV White E Barbour R Kozlov AP Heimer R Estimates of HIV incidence among drug users in St. Petersburg, Russia: continued growth of a rapidly expanding epidemic Eur J Public Health 2010 21 5 613 619 20798184 10.1093/eurpub/ckq115 PMC3180633

[B9] Verevochkin SV Shaboltas AV Gagarina SN Skochilov RV Toussova OV Krasnoselskih TV Malov SV Kozlov AP High HIV incidence rate in St. Petersburg IDU cohort Proceedings of the 6th IAS conference on HIV pathogenesis, treatment and prevention: 17–20 July 2011 2011 Rome, Italy: TUPE346

[B10] Wolfe D Paradoxes in antiretroviral treatment for injecting drug users: access, adherence and structural barriers in Asia and the former Soviet Union Int J Drug Policy 2007 18 4 246 254 10.1016/j.drugpo.2007.01.012 17689372

[B11] Krupitsky EM Zvartau EE Masalov DV Tsoi MV Burakov AM Egorova VY Didenko TY Romanova TN Ivanova EB Bespalov AY Verbitskaya EV Neznanov NG Grinenko AY O'Brien CP Woody GE Naltrexone for heroin dependence treatment in St. Petersburg, Russia J Subst Abuse Treat 2004 26 4 285 294 10.1016/j.jsat.2004.02.002 15182893

[B12] Amirkhanian YA Kelly JA Kuznetsova AV DiFranceisco WJ Musatov VB Pirogov DG People with HIV in HAART-era Russia: transmission risk behavior prevalence, antiretroviral medication-taking, and psychosocial distress AIDS Behav 2011 15 4 767 777 10.1007/s10461-010-9793-x 20803063 PMC4083488

[B13] WHO Regional Office for Europe WHO, HIV/AIDS treatment and care for injecting drug users 2006 Copenhagen: Clinical Protocol for the WHO European region

[B14] Harrigan PR Hogg RS Dong WW Yip B Wynhoven B Woodward J Brumme CJ Brumme ZL Mo T Alexander CS Montaner JS Predictors of HIV Drug-Resistance Mutations in a Large Antiretroviral-Naive Cohort Initiating Triple Antiretroviral Therapy J Infect Dis 2005 191 339 347 10.1086/427192 15633092

[B15] Bouhnik AD Chesney M Carrieri P Gallais H Moreau J Moatti JP Obadia Y Spire B MANIF 2000 Study Group Nonadherence among HIV-infected injecting drug users: the impact of social instability J Acquir Immune Defic Syndr 2002 31 Suppl 3 149 153 10.1097/00126334-200212153-00013 12562040

[B16] Hinkin CH Barclay TR Castellon SA Levine AJ Durvasula RS Marion SD Myers HF Longshore D Drug use and medication adherence among HIV-1 infected individuals AIDS Behav 2007 11 2 185 194 10.1007/s10461-006-9152-0 16897351 PMC2867605

[B17] Nolan S Milloy M-J Zhang R Kerr T Hogg RS Montaner JS Wood E Adherence and plasma HIV RNA response to antiretroviral therapy among HIV-seropositive injection drug users in a Canadian setting AIDS Care 2011 23 8 980 987 10.1080/09540121.2010.543882 21480010

[B18] Hicks PL Mulvey KP Chander G Fleishman JA Josephs JS Korthuis PT Hellinger J Gaist P Gebo KA HIV Research Network The impact of illicit drug use and substance abuse treatment on adherence to HAART AIDS Care 2007 19 9 1134 1140 10.1080/09540120701351888 18058397 PMC4126518

[B19] Werb D Mills EJ Montaner JS Wood E Risk of resistance to highly active antiretroviral therapy among HIV-positive injecting drug users: a meta-analysis Lancet Infect Dis 2010 10 7 464 469 10.1016/S1473-3099(10)70097-9 20610328 PMC13283459

[B20] Hesse M Vanderplasschen W Rapp R Broekaert E Fridell M Case management for persons with substance use disorders Cochrane Database Syst Rev 2007 4 CD006265 17943902 10.1002/14651858.CD006265.pub2

[B21] Vanderplasschen W Wolf J Rapp RC Broekaert E Effectiveness of different models of case management for substance-abusing populations J Psychoactive Drugs 2007 39 1 81 95 10.1080/02791072.2007.10399867 17523588 PMC1986794

[B22] Shelton RC Golin CE Smith SR Eng E Kaplan A Role of the HIV/AIDS case manager: analysis of a case management adherence training and coordination program in North Carolina AIDS Patient Care STDS 2006 20 3 193 204 10.1089/apc.2006.20.193 16548716

[B23] Katz MH Cunningham WE Fleishman JA Andersen RM Kellogg T Bozzette SA Shapiro MF Effect of case management on unmet needs and utilization of medical care and medications among HIV-infected persons Ann Intern Med 2001 135 8 Pt 1 557 565 11601927 10.7326/0003-4819-135-8_part_1-200110160-00006

[B24] Gardner LI Metsch LR Anderson-Mahoney P Loughlin AM del Rio C Strathdee S Sansom SL Siegal HA Greenberg AE Holmberg SD Antiretroviral Treatment and Access Study Study Group Efficacy of a brief case management intervention to link recently diagnosed HIV-infected persons to care AIDS 2005 19 4 423 431 10.1097/01.aids.0000161772.51900.eb 15750396

[B25] Kushel MB Colfax G Ragland K Heineman A Palacio H Bangsberg DR Case management is associated with improved antiretroviral adherence and CD4+ cell counts in homeless and marginally housed individuals with HIV infection Clin Infect Dis 2006 43 2 234 242 10.1086/505212 16779752

[B26] Malta M Carneiro-da-Cunha C Kerrigan D Strathdee SA Monteiro M Bastos FI Case management of human immunodeficiency virus-infected injection drug users: a case study in Rio de Janeiro, Brazil Clin Infect Dis 2003 37 Suppl 5 386 391 10.1086/377546 14648453

[B27] Cohen R Lynch S Bygrave H Eggers E Vlahakis N Hilderbrand K Knight L Pillay P Saranchuk P Goemaere E Makakole L Ford N Antiretroviral treatment outcomes from a nurse-driven, community-supported HIV/AIDS treatment programme in rural Lesotho: observational cohort assessment at two years J Int AIDS Soc 2009 12 23 10.1186/1758-2652-12-23 19814814 PMC2768674

[B28] Marazzi MC Bartolo M Emberti Gialloreti L Germano P Guidotti G Liotta G Magnano San Lio M Mancinelli S Modolo MA Narciso P Perno CF Scarcella P Tintisona G Palombi L Improving adherence to highly active anti-retroviral therapy in Africa: the DREAM programme in Mozambique Health Educ Res 2006 21 1 34 42 15947022 10.1093/her/cyh039

[B29] Remien RH Bastos FI Terto V Jr Raxach JC Pinto RM Parker RG Berkman A Hacker MA Adherence to antiretroviral therapy in a context of universal access, in Rio de Janeiro, Brazil AIDS Care 2007 19 6 740 748 10.1080/09540120600842516 17573593 PMC3539169

[B30] Siegal HA Li L Rapp RC Case management as a therapeutic enhancement: Impact on post-teatment criminality J Addict Dis 2002 21 4 37 46 10.1300/J069v21n04_04 12296500

[B31] Siegal HA Rapp RC Kelliher CW Fisher JH Wagner JH Cole PA The strengths perspective of case management: A promising inpatient substance abuse treatment enhancement J Psychoactive Drugs 1995 27 1 67 72 10.1080/02791072.1995.10471674 7602442

[B32] Vaughan-Sarrazin MS Hall JA Rick GS Impact of case management on use of health services by rural clients in substance abuse treatment J Drug Issues 2000 30 2 435 463

[B33] Menezes P Miller WC Wohl DA Adimora AA Leone PA Miller WC Eron JJ Jr Does HAART efficacy translate to effectiveness? Evidence for a trial effect PLoS One 2011 6 7 e21824 10.1371/journal.pone.0021824 21765918 PMC3135599

[B34] Krupitsky E Nunes EV Ling W Illeperuma A Gastfriend DR Silverman BL Injectable extended-release naltrexone for opioid dependence: a double-blind, placebo-controlled, multicentre randomised trial Lancet 2011 377 9776 1506 1513 10.1016/S0140-6736(11)60358-9 21529928

[B35] Zaller N Gillani FS Rich JD A model of integrated primary care for HIV-positive patients with underlying substance use and mental illness AIDS Care 2007 19 9 1128 1133 10.1080/09540120701335196 18058396

[B36] Bouis S Reif S Whetten K Scovil J Murray A Swartz M An integrated, multidimensional treatment model for individuals living with HIV, mental illness, and substance abuse Health Soc Work 2007 32 4 268 278 10.1093/hsw/32.4.268 18038728

[B37] Smith-Rohrberg D Mezger J Walton M Bruce RD Altice FL Impact of enhanced services on virologic outcomes in a directly administered antiretroviral therapy trial for HIV-infected drug users J Acquir Immune Defic Syndr 2006 43 Suppl 1 48 53 10.1097/01.qai.0000248338.74943.85 17133204

[B38] Lum PJ Tulsky JP The medical management of opioid dependence in HIV primary care settings Curr HIV/AIDS Rep 2006 3 4 195 204 10.1007/s11904-006-0016-z 17089480

